# Sex-related differences in blood concentrations and emergence profiles following total intravenous anesthesia with remimazolam and remifentanil

**DOI:** 10.1038/s41598-026-43531-7

**Published:** 2026-03-16

**Authors:** Riko Sato, Hitoshi Higuchi, Yukiko Nishioka, Saki Miyake, Takuya Miyawaki

**Affiliations:** 1 Department of Dental Anesthesiology and Special Care Dentistry, Okayama University Graduate School of Medicine, Dentistry and Pharmaceutical Sciences, Okayama, Japan; 2 Department of Dental Anesthesiology, Okayama University Hospital, Okayama, Japan

**Keywords:** Medical research, Neuroscience

## Abstract

Remimazolam is a novel, short-acting benzodiazepine, which is characterized by rapid onset and quick recovery. The clinical efficacy and metabolism of many intravenous anesthetics are known to be influenced by sex; however, the effects of sex on the anesthetic efficacy and metabolism of remimazolam remain unclear. This prospective observational study examined sex-related differences in pharmacokinetics and emergence profiles after total intravenous anesthesia was induced with remimazolam and remifentanil in patients undergoing oral and maxillofacial surgery. Thirty-five American Society of Anesthesiologists Physical Status 1 adults (19 females, 16 males), aged 18–49 years, received standardized dosing based on their actual body weights. Serum remimazolam concentrations were measured at the end of administration and immediately before extubation using high-performance liquid chromatography. Although the emergence time did not differ significantly between the sexes, the mean emergence time of the females was approximately 80 s shorter. Serum remimazolam concentrations were significantly lower in females at both measurement time points (*p* < 0.001). This may suggest that remimazolam is metabolized more rapidly in women. Although these sex-related pharmacokinetic differences did not affect the time to awakening under combined remimazolam and remifentanil anesthesia, clinicians should be aware of potential sex differences in the pharmacokinetics of remimazolam.

**Supplementary Information:**

The online version contains supplementary material available at 10.1038/s41598-026-43531-7.

## Introduction

Remimazolam is a novel intravenous anesthetic belonging to the benzodiazepine class. Unlike conventional benzodiazepines, it is not metabolized by hepatic cytochrome P450 enzymes. Instead, it undergoes rapid hydrolysis by hepatic carboxylesterases into inactive metabolites. This unique metabolic pathway contributes to its rapid onset of action and swift recovery profile. Compared to propofol, remimazolam is associated with reduced cardiovascular and respiratory depressive effects, which may enhance patients’ hemodynamic stability during anesthesia. Furthermore, the availability of a specific antagonist, flumazenil, provides an additional safety advantage, as it allows prompt antagonism of the sedative effects of remimazolam if needed. These features make remimazolam a promising anesthetic agent, with the potential for widespread use in clinical practice.

To ensure the safe management of intravenous anesthesia, it is essential to have a clear and comprehensive understanding of the pharmacokinetic properties of intravenous anesthetic agents. Previous studies have demonstrated that commonly used agents, such as midazolam and propofol, exhibit sex-based differences in their pharmacodynamic effects^1–4^. For example, females tend to recover more rapidly from propofol-induced anesthesia than males.

In the case of remimazolam, pharmacokinetic simulations have suggested that females may require doses that are 10–20% higher than those administered to males to achieve equivalent anesthetic effects^5^. This implies that, like other intravenous anesthetics, remimazolam may also exhibit sex-related differences in anesthetic sensitivity and pharmacodynamics. Although some studies have explored potential sex-related differences in the effects of remimazolam, the evidence remains inconclusive^6,7^. Moreover, no previous studies have systematically investigated sex-based differences in remimazolam’s effects in direct relation to its pharmacokinetic profile. Further research is therefore warranted to elucidate the influence of sex on both the pharmacokinetics and pharmacodynamics of remimazolam, which may help to optimize individualized dosing strategies and enhance the safety and efficacy of anesthesia.

In this study, we investigated the influence of sex-based differences on emergence from total intravenous anesthesia (TIVA) induced with remimazolam in combination with remifentanil. The primary objective was to assess whether the anesthetic effects of remimazolam differ between males and females. To further explore the mechanisms underlying any such differences, we also examined the possibility of potential sex-related differences in the pharmacokinetics of remimazolam by measuring its serum concentration after anesthesia. Thus, this study aimed to increase our understanding of sex-based variability in anesthetic responses and support more individualized anesthetic management.

## Methods

This prospective observational study was conducted after approval had been given by Okayama University Graduate School of Medicine, Dentistry and Pharmaceutical Sciences and Okayama University Hospital, Ethics Committee (approval No. 2305-022), and was registered with the University Medical Information Network (UMIN) Clinical Trials Registry (UMIN000051489). We have read the Helsinki Declaration and have followed its guidelines in this study. Written informed consent for participation in the study was obtained from all participants.

### Subjects

The study included patients who were scheduled to undergo oral and maxillofacial surgery under general anesthesia induced with remimazolam and remifentanil and managed by the Department of Dental Anesthesiology at Okayama University Hospital between May 2023 and March 2025.

The inclusion criteria were as follows: (1) the ability to provide written informed consent; (2) being aged between 18 and 49 years; (3) a body mass index (BMI) of < 30; (4) an American Society of Anesthesiologists Physical Status (ASA-PS) classification of PS-1; (5) an anticipated anesthesia duration of less than 4 h; (6) undergoing minimally invasive oral surgery, such as dental procedures or tooth extraction; and (7) not requiring opioid analgesics for postoperative pain management. The exclusion criteria were as follows: (1) known contraindications for remimazolam, remifentanil, or rocuronium; (2) a requirement for premedication; and (3) determination by the principal investigator or sub-investigators that the patient’s participation was inappropriate.

As no prior data were available to support a formal power calculation for awakening time, this study was designed as an exploratory prospective observational study. Accordingly, no formal hypothesis-testing–based power calculation was performed. The study population consisted of all subjects who met the inclusion criteria, did not meet any exclusion criteria, and provided written informed consent during the study period.

### Anesthetic management and blood sampling

Standard monitoring included non-invasive blood pressure (NBP), electrocardiography (ECG), peripheral oxygen saturation (SpO₂), end-tidal CO₂ (ETCO₂), and the bispectral index (BIS). General anesthesia was induced and maintained with remimazolam and remifentanil-induced TIVA. All anesthesia data, including BIS values, were automatically recorded using the Prescient^®^ OR anesthesia management system (Fujifilm Medical Co., Tokyo).

For induction, remimazolam was administered at 12 mg/kg/h and remifentanil was administered at 0.3–0.5 µg/kg/min using syringe pumps. After confirming the loss of consciousness, the remimazolam infusion rate was reduced to 1 mg/kg/h. Rocuronium (0.6–0.9 mg/kg) was administered intravenously to facilitate tracheal intubation.

Anesthesia was maintained with remimazolam at approximately 1 mg/kg/h, with the dose adjusted by the attending dental anesthesiologist, based on the patient’s clinical signs and BIS values. If signs of arousal appeared, an additional 5 mg bolus of remimazolam was administered, as needed. Remifentanil was similarly adjusted within the range of 0.1–0.5 µg/kg/min, depending on surgical stimulation.

Prior to completion of the procedure, dedicated venous access for blood collection was secured in the upper limb contralateral (median cubital vein or cephalic vein) to the remimazolam administration site. Following completion of the procedure, 10 ml of blood was collected for measurement of remimazolam blood concentration. Thereafter, remimazolam and remifentanil were discontinued to allow the patient to emerge from the anesthesia. If residual neuromuscular blockade was suspected based on neuromuscular monitoring, sugammadex was administered.

Postoperative analgesia was managed using NSAIDs alone, which were administered as needed. No arousal stimuli were administered for 10 min after discontinuation of remimazolam and remifentanil. After this period, verbal calls and gentle tapping on the shoulder were applied every 2 min, with no other stimuli used. The criteria for extubation included: (1) spontaneous respiration, (2) eye opening, (3) response to verbal commands, and (4) the presence of a cough reflex. Extubation was performed by the attending dental anesthesiologist once all the criteria were met. These criteria were applied uniformly to all patients according to a standardized protocol. Immediately prior to extubation, a second 10 mL blood sample was collected from the venous access line for blood collection and used to measure the remimazolam concentration. If the patient did not regain consciousness within 30 min of remimazolam being discontinued, an additional 10 mL blood sample was taken, and flumazenil was administered as an antagonist.

### Outcome measures

The primary outcome was the time from the discontinuation of remimazolam to extubation (the emergence time). The secondary outcomes included the remimazolam serum concentrations at the time of remimazolam discontinuation and at extubation (or at the time of flumazenil administration, if applicable). To account for differences in administered dose, dose-normalized remimazolam concentrations were also calculated by dividing the measured serum concentration by the mean administered dose (mg/kg). BIS values were recorded at the following timepoints: before anesthesia, at the loss of consciousness, at the end of remimazolam administration, and at extubation. Additional outcomes included the time from the start of remimazolam administration to the loss of consciousness (the induction time), the total and mean doses of remimazolam and remifentanil, and their administration rates. Patient demographic data, including age, body weight, height, BMI, and sex, as well as surgical information, were extracted from the anesthesia records. These outcomes were compared between men and women.

### Measurement of the remimazolam concentration

1. Creation of calibration curves

First, we prepared standard solutions of remimazolam-containing serum to create a remimazolam calibration curve. Raw remimazolam powder (provided free of charge by Mundipharma K.K.) was diluted to concentrations of 2, 5, 10, 20, and 40 µg/mL using a 50% ethanol solution (ethanol: distilled water = 1:1). To prepare serum samples, 100 µL of each diluted remimazolam solution was mixed with 1000 µL of pooled human serum prepared from volunteers.

The method for measuring the blood concentration of remimazolam was based on our previous method^8–10^ and involved remimazolam being extracted from serum samples using a liquid-liquid extraction method. To 1100 µL of sample, 100 µL of distilled water, 1 mL of 0.1 M dipotassium hydrogen phosphate, 100 µL of internal standard (IS) (10 µg/mL diazepam), and 5 mL of diethyl ether were added. The mixture was shaken at 120 rpm for 10 min and centrifuged at 3,000 rpm for 10 min, and then the ether layer was collected. After adding 4 mL of distilled water, the mixture was shaken again for 10 min at 120 rpm and centrifuged again. The ether layer was collected and evaporated to dryness. The residue was reconstituted in 180 µL of a solution containing 50% ethanol, acetonitrile, and 0.01 M dipotassium hydrogen phosphate (2:9:9), and 50 µL was injected into a high-performance liquid chromatography (HPLC) column.

The mobile phase in the HPLC system consisted of distilled water, acetonitrile, and 0.25 M potassium dihydrogen phosphate (pH 4.2–4.6) (67.2: 30.0: 2.8 (v/v/v)). The flow rate was 1.0 mL/min, and separation was achieved at 40℃ with a TSKgel^®^ ODS-80Ts QA (5 μm) column (internal diameter: 4.6 mm × length: 15 cm; Tosoh Corporation, Tokyo, Japan). The mobile phase was monitored using a UV detector (SPD-20 A; Shimazu Co., Tokyo, Japan) at wavelength of 214 nm. The peak-area ratio of remimazolam to the IS was obtained from the HPLC chromatogram at each concentration, and the ratio of the peak area of remimazolam to the peak area of the IS (y) was plotted against the known concentration (x), and linear regression analysis was performed using the least-squares method to create a calibration curve for remimazolam (Supplementary Fig. 1).

2. Measurement of the remimazolam concentrations of blood samples

Blood samples were allowed to stand at room temperature for 30 min and subsequently centrifuged at 2,000 G for 10 min to separate the serum, which was then stored at -4 °C. The remimazolam concentration was determined using the abovementioned procedure, with quantification performed based on the obtained calibration curve.

### Statistical analysis

Statistical analyses were conducted using t-tests or Mann-Whitney U tests in GraphPad Prism^®^, version 10 (GraphPad Software, Inc., San Diego, USA). Bivariate correlation analyses were also performed to assess the association between awake time and each predictor individually using Pearson’s correlation coefficient. Results with p-values of < 0.05 were considered significant, and all results are presented as mean ± standard deviation values.

## Results

**Fig. 1:**
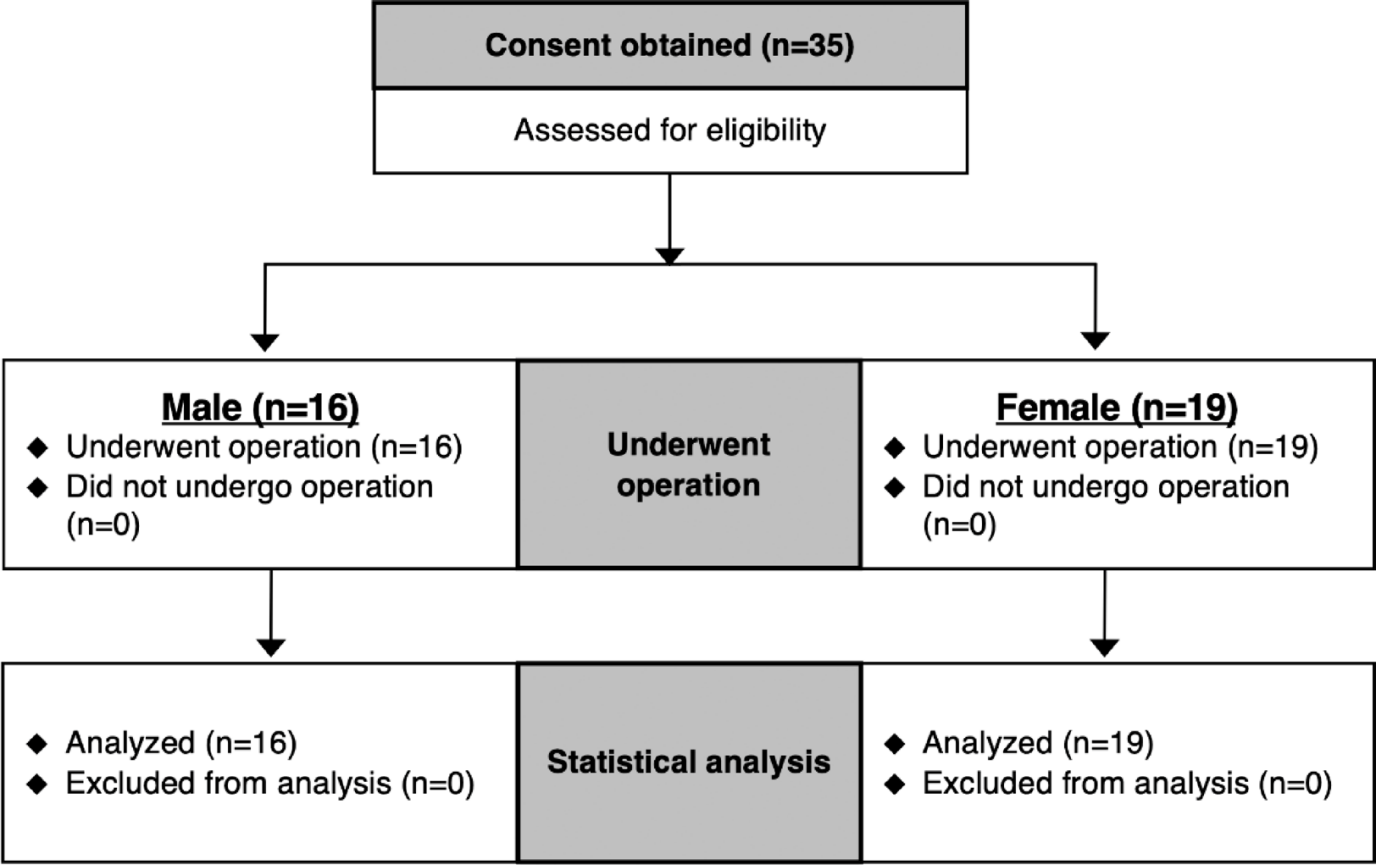
Flow diagram of the study design.

Figure 1 shows a flow diagram of the participant enrollment process for this study. A total of 19 female and 16 male participants were included. No participants dropped out during the surgical procedures or subsequent analyses.

The demographic and clinical characteristics of each group are summarized in Table 1. There were no significant differences in age. Height and weight were significantly higher in males; however, there was no difference in BMI between the two groups. The operative time did not differ significantly between the sexes, and the surgical procedures were comparable. There was no patient who was administered flumazenil.

**Table 1:** Patients’ background, clinical, and anesthesia characteristics.

	Male (*n* = 16)	Female (*n* = 19)	*P*-value
Age	27.4 ± 9.5	31.5 ± 9.1	0.20
Height (cm)	172.0 ± 5.9	157.1 ± 4.9	< 0.0001
Weight (kg)	64.8 ± 9.5	51.4 ± 5.5	< 0.0001
BMI (kg/m^2^)	21.9 ± 3.3	20.9 ± 2.4	0.27
Operation time (min)	86.9 ± 41.9	89.3 ± 40.0	0.868
Type of operation (n)			
Tooth extraction	6	5	
Tooth extraction and cystectomy	2	3	
Cystectomy	1	0	
Plate removal	4	7	
Plate removal and genioplasty	1	4	
Plate removal and tooth extraction	2	0	
Remimazolam			
Administration time (min)	119.9 ± 44.9	123.2 ± 44.5	0.83
Total dose (mg)	152.9 ± 47.0	115.8 ± 32.1	0.0093
Mean dose (mg/kg)	2.38 ± 0.79	2.27 ± 0.68	0.66
Administration rate (mg/kg/h)	1.21 ± 0.097	1.14 ± 0.137	0.090
Remifentanil			
Administration time (min)	120.6 ± 44.8	123.7 ± 43.9	0.837
Total dose (mg)	1.57 ± 0.68	1.21 ± 0.49	0.073
Mean dose (µg/kg)	24.9 ± 12.6	23.8 ± 10.5	0.780
Administration rate (µg/kg/min)	0.20 ± 0.033	0.191 ± 0.035	0.323
Fulmazenil administration (n)	0	0	

Mean ± SD. BMI: Body mass index.

The total doses of both remimazolam and remifentanil were higher in males, probably due to differences in body weight. However, there were no significant differences in the mean dose or administration rate of either drug.

Table 2 shows the time to the loss of consciousness and the BIS values before anesthesia and at the time of the loss of consciousness. No significant differences were observed between the sexes in either the time to the loss of consciousness or the BIS values at each timepoint.

**Table 2:** Time of loss of consciousness and BIS values at anesthesia induction.

	Male (*n* = 16)	Female (*n* = 19)	Mean difference (Female -Male) (95%CI)	*P*-value
Time of loss of consciousness (sec)	93.1 ± 20.7	91.9 ± 17.8	– 1.2 (– 14.5 to 12.0)	0.851
BIS value				
Pre-anesthesia	94.9 ± 3.7	95.4 ± 3.5	0.54 (– 1.94 to 3.03)	0.657
Loss of consciousness	68.8 ± 12.5	71.3 ± 9.9	2.6 (– 5.13 to 10.3)	0.503

Mean ± SD. BIS Bispectral inex, CI Confidence interval.

The emergence time, the BIS values at the end of remimazolam administration and at the time of extubation, and the blood concentrations of remimazolam at the same timepoints are presented in Table 3. Women awakened on average 80 s faster than men, however, no significant intersex differences were observed in the emergence time or the BIS values at each timepoint. In contrast, the remimazolam blood concentrations at the end of administration and at the time of endotracheal tube removal were significantly lower in females (*P* < 0.0001 and *P* = 0.0003, respectively). Even after dose normalization, remimazolam concentrations at both the end of administration and at extubation remained significantly lower in females than in males (*P* = 0.0075 and *P* = 0.028, respectively).

**Table 3:** Awake time, BIS value, and remimazolam concentration at emergence from anesthesia.

	Male (*n* = 16)	Female (*n* = 19)	Mean difference (Female -Male) (95%CI)	*P*-value
Emergence time (sec)	785.4 ± 234.7	701.8 ± 253.5	– 83.6 (– 252.8 to 85.6)	0.32
BIS value				
End of remimazolam administration	60.0 ± 7.8	62.0 ± 9.6	2.0 (– 4.11 to 8.11)	0.51
Extubation	78.0 ± 6.4	76.3 ± 5.9	– 1.68 (– 5.93– 2.56)	0.43
Remimazolam concentration (ng/mL)				
End of remimazolam administration	1311 ± 201	882 ± 272	– 429.0 (– 596.3 to – 261.6)	< 0.0001
Extubation	746 ± 148	548 ± 144	– 198.3 (– 298.8 to – 97.7	0.0003
Dose-normalized remimazolam concentration				
End of remimazolam administration	601.8 ± 200.1	420.8 ± 176.1	– 181.1 (– 310.4 to – 51.7)	0.0075
Extubation	334.5 ± 91.9	259.8 ± 99.2	– 74.7 (– 141.0 to – 8.5)	0.028

Mean ± SD. BIS: Bispectral Index, CI Confidence interval.

The bivariate correlations between the examination outcomes and the emergence times of all participants are shown in Table 4. In the bivariate regression analyses, only the BIS value at the end of remimazolam administration was significantly correlated with the emergence time.

**Table 4:** Bivariate correlation analysis between the examined predictors and the awake time.

		Correlation coefficient	95% confidence interval	*P*-value
Age		0.05924	– 0.2795 to 0.3849	0.7353
Height		– 0.03387	– 0.3630 to 0.3028	0.8468
Weight		– 0.01285	– 0.3446 to 0.3218	0.9416
BMI		– 0.002368	– 0.3354 to 0.3311	0.9892
BIS value	End of remimazolam administration	– 0.4958	– 0.7115 to -0.1948	0.0025
	Extubation	– 0.02524	– 0.3555 to 0.3106	0.8856
Remimazolam concentration	End of remimazolam administration	0.2417	– 0.09953 to 0.5321	0.1618
	Extubation	– 0.04808	– 0.3753 to 0.2898	0.7839
Remimazolam	Administration time	– 0.2378	– 0.5291 to 0.1036	0.1689
	Total dose	– 0.2692	– 0.5528 to 0.07040	0.1179
	Mean dose	– 0.2845	– 0.5643 to 0.05383	0.0976
	Administration rate	– 0.03450	– 0.3636 to 0.3022	0.8440
Remifentanil	Administration time	– 0.2323	– 0.5249 to 0.1094	0.1793
	Total dose	– 0.07829	– 0.4011 to 0.2618	0.6548
	Mean dose	– 0.07669	– 0.3997 to 0.2633	0.6615
	Administration rate	0.2012	– 0.1415 to 0.5009	0.2464

BMI body mass index, BIS bispectral index.

## Discussion

This cohort study demonstrated sex-based differences in the pharmacokinetics of remimazolam. Using HPLC to measure blood concentrations, we found that females had significantly lower serum levels of remimazolam than males, despite receiving the same actual body weight-based dosing regimen. Although there was a trend toward shorter emergence times in females following remimazolam and remifentanil-induced TIVA, the difference was not significant.

Previous studies have suggested the existence of sex-based differences in remimazolam pharmacokinetics. Pharmacokinetic modeling studies based on data from previous clinical trials have consistently shown higher remimazolam clearance in females than in males^5,11,12^. Masui et al. demonstrated that female patients required infusion rates 10% to 20% higher than those of male patients to maintain equivalent remimazolam concentrations^5^. In our study, when a standardized dosing regimen was used in both sexes, the blood concentrations of remimazolam at the end of administration and at the time of endotracheal tube removal were both significantly lower in females. These findings corroborate earlier reports of sex-specific variations in remimazolam pharmacokinetics in studies involving simulation models. One possible reason for the lower remimazolam levels seen in females is differences in carboxylesterase activity. Unlike traditional benzodiazepines, which are metabolized by hepatic CYP enzymes, remimazolam features a unique ester bond that undergoes rapid hydrolysis by tissue esterases, primarily carboxylesterase 1 (CES1) in the liver^13^. CES1 activity tends to be higher in females^14,15^, potentially leading to faster remimazolam metabolism. Another contributory factor to remimazolam blood levels is the disparity in body weight between males and females. In our study, the males exhibited significantly greater height and weight, which was reflective of the inherent biological differences between the sexes. While current clinical practice generally bases continuous remimazolam dosing on actual body weight, Masui et al. proposed adjusting dosages according to ideal body weight in their pharmacokinetic model for general anesthesia. Their research indicates that using actual body weight may result in higher remimazolam concentrations in heavier patients than in lighter patients when dosing is performed uniformly per kilogram^5^. Given our adherence to actual body weight for dosing, the observed sex-related differences in body weight likely contributed to the higher remimazolam blood levels observed in males. However, the persistence of lower dose-normalized concentrations in females suggests that factors beyond simple dosing differences may contribute to sex-related variability in remimazolam pharmacokinetics.

In the current study, we found that remimazolam concentrations were significantly lower in females than in males. However, there was no significant difference in the emergence time between the two groups. Several previous studies have investigated the factors that influence the emergence time following remimazolam-induced anesthesia. For example, Lohmer et al. examined predictors of the extubation time in a clinical study^16^. Their findings indicated that the final infusion rate of remimazolam, the BIS score at the end of infusion, and sex were significant factors affecting the extubation time. Notably, females exhibited a 3- to 5-minute shorter extubation time than males. In contrast, Shimamoto et al.^17^ categorized patients into two groups based on an extubation time cut-off value of 15 min: the short- and long-period groups. They analyzed the factors that contributed to prolonged extubation in the long-period group and found that the patients in this group had significantly higher BMI and significantly lower albumin concentrations and were significantly older than those in the short-period group. In the latter study, sex was not identified as a significant factor influencing the extubation time.

In the present study, the mean emergence time of the females was approximately 80 s shorter than that of the males. Although no significant sex-related difference was observed in emergence time, this endpoint represents a composite clinical measure influenced by multiple factors, including airway reflex recovery and clinical judgment, and should therefore be interpreted cautiously as a surrogate of anesthetic efficacy. Especially, it is important to note that in our study general anesthesia was maintained with a combination of remimazolam and remifentanil, both of which were discontinued simultaneously at the end of surgery. Therefore, it is likely that remifentanil also contributed to the observed emergence times, making it difficult to interpret the results based solely on remimazolam concentrations.

Opioids, such as remifentanil, are known to suppress airway reflexes, including the cough reflex elicited by airway devices, such as endotracheal tubes^18^. Previous studies have reported sex-related differences in the effect-site concentration of remifentanil required to suppress the cough reflex during extubation, with males requiring higher concentrations than females^19–21^. In other words, the return of the cough reflex during anesthetic awakening, which is essential for safe extubation, may occur earlier in males. In our study, the presence of an adequate cough reflex was one of the criteria for proceeding with extubation. Thus, it is highly plausible that the difference in remifentanil sensitivity between the sexes influenced the emergence time, potentially masking the effect of the remimazolam concentration alone.

Indeed, in our study, neither the remimazolam blood concentration at the end of administration nor that at the time of extubation was significantly correlated with the emergence time. The only parameter that showed a significant correlation with the emergence time was the BIS value at the end of remimazolam administration. These findings suggest that the emergence time following remimazolam and remifentanil-induced anesthesia is probably determined by multiple factors, including remimazolam pharmacokinetics, opioid effects, and patient-specific sensitivity to these agents. In this study, had we individually assessed the times of our four extubation criteria, we might have been able to consider them as supplementary. These evaluations may have provided greater objectivity than the subjective overall assessments by dental anesthesiologists. However, in this study, these specific points in time were not recorded individually. Further detailed investigations are necessary to better understand the complex interplay of these variables and to refine individualized anesthesia strategies.

Our study had several limitations. First, no formal sample size calculation was performed prior to study initiation because of the lack of prior data on awakening time, and the study was therefore designed as an exploratory observational study. As a result, the study may have been underpowered to detect small but clinically relevant differences. Accordingly, the results should be interpreted with caution, with emphasis placed on the observed mean difference (effect sizes) and their confidence intervals rather than on statistical significance alone.

Second, although we observed sex-based differences in anesthesia sensitivity, we did not investigate the potential influence of sex hormones or the menstrual cycle in the female participants. Previous studies have suggested that hormonal fluctuations, particularly testosterone and progesterone levels, may affect anesthetic sensitivity. For example, Erden et al.^22^ reported that the minimum alveolar concentration of sevoflurane was decreased during the luteal phase of the menstrual cycle, when progesterone levels are elevated. In animal studies, Wasilczuk et al.^23^ demonstrated that castrated male mice exhibited reduced sensitivity to isoflurane, while testosterone replacement restored their anesthetic responsiveness. More recently, sex hormones have been shown to modulate GABA_A_ receptors, which are the primary target of many anesthetic agents, including remimazolam. Our study population ranged in age from 18 to 50 years, making it likely that hormone levels varied among individuals within the same sex. However, we did not collect data on menstrual cycle phase, hormonal status, or the use of hormonal contraceptives, all of which may influence anesthetic pharmacodynamics. Future studies incorporating hormonal profiling or menstrual cycle tracking in female participants would be valuable to further elucidate these potential effects.

Third, all of the participants in our study were Japanese. Therefore, the generalizability of our findings to other ethnic groups and geographic regions may be limited. CES1, the primary enzyme responsible for remimazolam metabolism, exhibits known genetic polymorphisms that can affect enzymatic activity and drug metabolism^24–26^. These CES1 polymorphisms have been shown to influence remimazolam clearance^13^. Moreover, the frequency of CES1 polymorphisms varies among different racial and ethnic populations^27–29^, raising the possibility of racial or ethnic differences in remimazolam pharmacokinetics. As such, the generalizability of our findings to non-Japanese populations remains uncertain. Future studies in diverse populations are necessary to confirm whether our results can be extrapolated beyond Japanese subjects.

Fourth, our study was limited by the small number of blood sampling timepoints (only two), which precluded the calculation of detailed pharmacokinetic parameters, such as clearance, volume of distribution, and elimination half-life values. Despite weight-based dosing, the significantly lower serum concentrations of remimazolam observed in women, along with lower dose-normalized concentrations, suggest a potential gender difference in the pharmacokinetics of remimazolam. However, since formal pharmacokinetic parameters such as clearance were not estimated in this study, these findings should be interpreted as hypothesis-generating rather than definitive evidence of faster metabolic rates in women. Theoretically, pharmacokinetic parameter estimation after discontinuation of a constant-rate intravenous infusion at steady state requires a minimum of three concentration measurements: one at the end of infusion to determine Css and at least two during the elimination phase to estimate the elimination rate constant. However, because estimation based on only two elimination-phase points is sensitive to variability, four to five sampling points are generally considered appropriate in practice to ensure robust parameter estimation^30–32^. A more comprehensive sampling schedule would allow for full pharmacokinetic modeling and a better understanding of sex differences in remimazolam metabolism. Therefore, future studies using this dataset as preliminary data, but incorporating more frequent sampling and advanced pharmacokinetic analysis, are warranted.

In conclusion, this study is the first to investigate sex-related differences in the pharmacokinetics of remimazolam by directly measuring serum concentrations using HPLC. We evaluated the differences in both the emergence time and remimazolam blood concentrations between male and female patients in whom TIVA was induced with a combination of remimazolam and remifentanil. Our findings demonstrated that, although there was no significant difference in the emergence time between the sexes, female patients exhibited significantly lower serum remimazolam concentrations than male patients under an identical weight-based dosing regimen. Although these findings align with previous reports suggesting sex-related differences in remimazolam clearance, the present data do not allow definitive conclusions regarding metabolic rate, and further studies incorporating detailed pharmacokinetic analyses are warranted.

Future studies with more intensive sampling and mechanistic approaches are needed to clarify the underlying causes of these sex-related differences and to develop dosing strategies that better account for individual patient characteristics, including sex, hormonal status, and genetic polymorphisms. strategies that account for individual patient characteristics, including sex, hormone status, and genetic polymorphisms.

## Supplementary Information

Below is the link to the electronic supplementary material.

Supplementary Material 1

## Data Availability

The datasets generated during and/or analyzed during the current study are available from the corresponding author on reasonable request.
