# Supplementary material for: Sex-related differences in blood concentrations and emergence profiles following total intravenous anesthesia with remimazolam and remifentanil
Source: Sci Rep. 2026 Mar 16;16:13650. doi: 10.1038/s41598-026-43531-7 (PMC13125501; doi:10.1038/s41598-026-43531-7)
Supplement: Supplementary file 1 — Supplementary Material 1 [file 41598_2026_43531_MOESM1_ESM.pptx]

## Slide 1
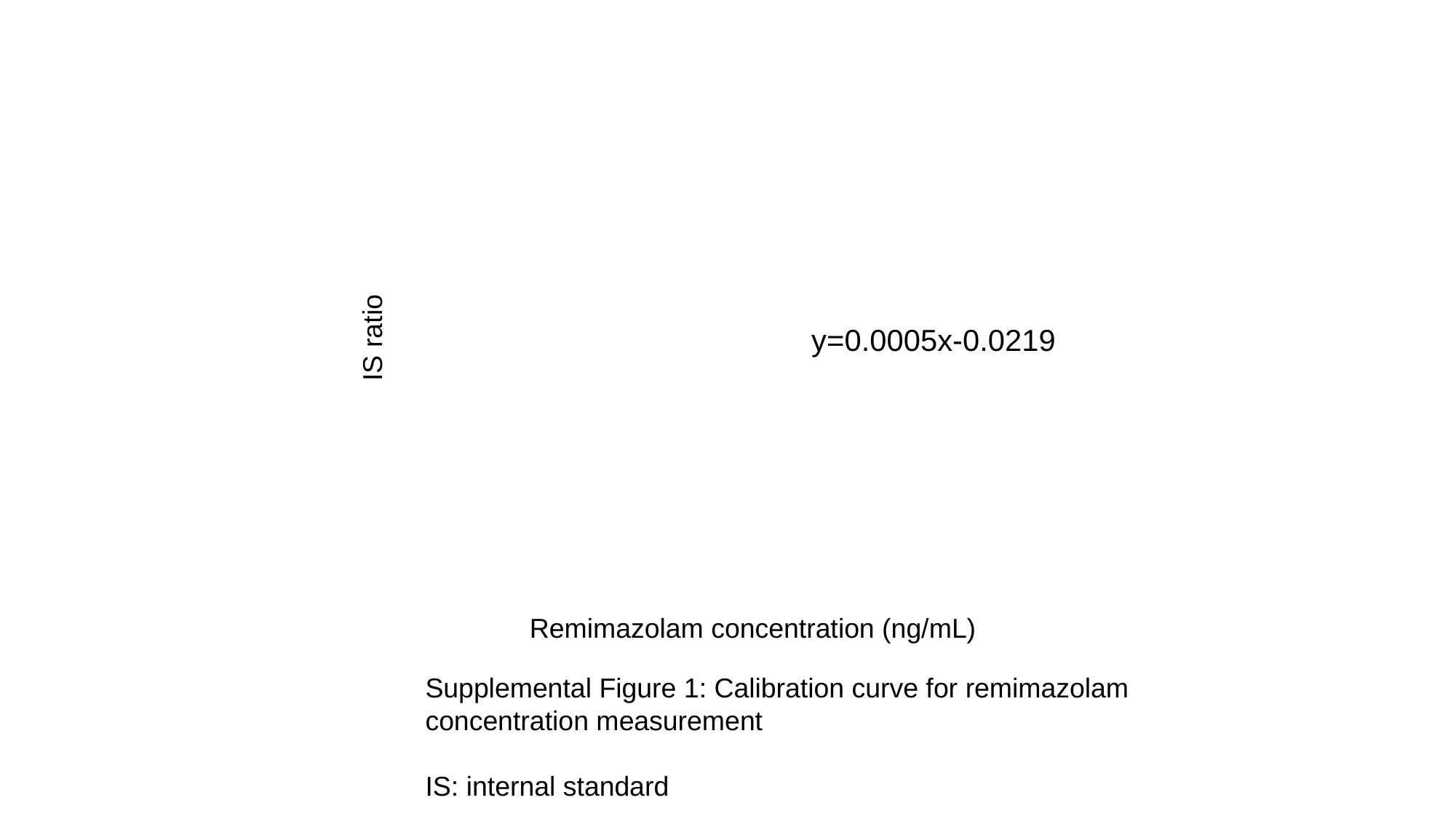

IS ratio
y=0.0005x-0.0219
Remimazolam concentration (ng/mL)
Supplemental Figure 1: Calibration curve for remimazolam concentration measurement
IS: internal standard
